# Optical prediction of single muscle fiber force production using a combined biomechatronics and second harmonic generation imaging approach

**DOI:** 10.1038/s41377-018-0080-3

**Published:** 2018-10-24

**Authors:** Dominik Schneidereit, Stefanie Nübler, Gerhard Prölß, Barbara Reischl, Sebastian Schürmann, Oliver J Müller, Oliver Friedrich

**Affiliations:** 1 Institute of Medical Biotechnology, Friedrich-Alexander-University (FAU) Erlangen-Nürnberg, Paul-Gordan-Str. 3, 91052 Erlangen, Germany; 2 Erlangen Graduate School in Advanced Optical Technologies (SAOT), FAU Erlangen-Nürnberg, Paul-Gordan-Str. 7, 91052 Erlangen, Germany; 3 Muscle Research Center Erlangen (MURCE), Friedrich-Alexander-University Erlangen-Nürnberg, Erlangen, Germany; 4 Department of Internal Medicine III, University of Kiel, Arnold-Heller-Str. 3, 24105 Kiel, Germany; 5 DZHK (German Center for Cardiovascular Research) Partner Site Hamburg/Kiel/Lübeck, Kiel, Germany

## Abstract

Skeletal muscle is an archetypal organ whose structure is tuned to match function. The magnitude of order in muscle fibers and myofibrils containing motor protein polymers determines the directed force output of the summed force vectors and, therefore, the muscle’s power performance on the structural level. Structure and function can change dramatically during disease states involving chronic remodeling. Cellular remodeling of the cytoarchitecture has been pursued using noninvasive and label-free multiphoton second harmonic generation (SHG) microscopy. Hereby, structure parameters can be extracted as a measure of myofibrillar order and thus are suggestive of the force output that a remodeled structure can still achieve. However, to date, the parameters have only been an indirect measure, and a precise calibration of optical SHG assessment for an exerted force has been elusive as no technology in existence correlates these factors.  We engineered a novel, automated, high-precision biomechatronics system into a multiphoton microscope allows simultaneous isometric Ca^2+^-graded force or passive viscoelasticity measurements and SHG recordings. Using this *MechaMorph* system, we studied force and SHG in single EDL muscle fibers from wt and *mdx* mice; the latter serves as a model for compromised force and abnormal myofibrillar structure. We present Ca^2+^-graded isometric force, pCa-force curves, passive viscoelastic parameters and 3D structure in the same fiber for the first time. Furthermore, we provide a direct calibration of isometric force to morphology, which allows noninvasive prediction of the force output of single fibers from only multiphoton images, suggesting a potential application in the diagnosis of myopathies.

## New Biomechatronics-Multiphoton Microscopy predicts the degree of muscle weakness from the cellular structure in 3D

A laser-scanning multiphoton microscope that diagnoses cellular-level structural defects in skeletal muscle in 3D has been combined with a miniaturized biomechatronics diagnostic machine developed by German researchers. Structural changes to muscle cells, such as the branching and splitting of individual fibers, or subcellular remodeling of cellular skeleton, have been associated with degenerative ailments in patients. Now, Oliver Friedrich at the Friedrich-Alexander-University in Erlangen and colleagues have developed a technique that links the force a muscle cell can apply with its cellular architecture. The team used a special version of multiphoton microscopy, Second Harmonic Generation, to examine single muscle fibers extracted from wild type mice or muscular dystrophy ones carrying a mutation in the dystrophin gene. Simultaneously, an automated system measures the mechanical properties of contractions induced by defined concentrations of calcium ions, or how fibers react to passive stress. Computer analysis yielded linear correlations between optical images and muscle function that may be useful in predicting biomechanical disease in patients from optical assessment of patient biopsies using multiphoton imaging.

## Introduction

Structure and function are inevitably related to each other. A desired function requires a tailored structure, while from a given structure, deductions regarding its function may be derived. This particularly applies to the concept of organ and tissue structures and functions. Skeletal muscle, for instance, is highly ordered and hierarchically structured by parallel and serial polymeric motor proteins, i.e., actomyosin filaments, and series elastic elements, to perform as a linear bioactuator to enable movement and to give in passively to external forces^1^. Within each muscle fiber, the concept of highly ordered elements, i.e., myofibrils aligned in parallel by sarcomeric and extrasarcomeric proteins at the z-disks^2^, ensures the well-known striation-pattern^3^ and directs force production along the main fiber axis^4^. Thus, apart from fast signaling-related activation processes that determine muscle performance, e.g., excitation contraction coupling and Ca^2+^ homeostasis^5^, a long-term predictor of muscle function is found in structural changes. This is particularly important as skeletal muscle has a high plasticity to respond to exercise with hypertrophy, to disuse with atrophy^6^, and to injury with complete regeneration and repair^7^. Similarly, muscle structure and cytoarchitecture are also major targets in muscle diseases, such as chronic inflammatory or degenerative diseases associated with ongoing degeneration-regeneration cycles, imperfect repair, and tissue and cellular remodeling. Examples include (poly/dermato-)myositis^8^, cancer cachexia^9^, muscular dystrophies^10^, and even aging^11^. Muscle structure remodeling involves not only the extracellular matrix (e.g., fibrosis) but also the remodeling of the sarcomere and the myofibrillar cytoarchitecture. For instance, a hallmark of structural cellular changes as a result of regeneration has been found in single fiber branching and splitting, for example, in Duchenne muscular dystrophy^12–14^, following weight-lift exercise^15^ or toxin-induced myonecrosis^16^. Thus, it is not surprising that chronic conditions with ongoing remodeling are associated with progressive muscle weakness^12,17^. Therefore, the three-dimensional sterical arrangement of myofibrillar and cytoarchitecture may be considered an anatomical correlate to predict muscle force outcomes. It is tempting to speculate that imaging of the 3D muscle fiber architecture can be a modality for extrapolating mechanical performance instead of executing strenuous force recordings using force transducer technologies, in particular in cases involving single fibers. However, the assessment of skeletal muscle fiber architecture is not straightforward. Clinically, the diagnosis of myopathy in patients presenting muscle weakness is usually based on histology section analysis from muscle biopsies. Although cross-cut sections allow visualization of multiple cells at once, this approach is limited to one plane without any 3D aspect. Alternatively, confocal laser scanning microscopy has frequently been used to determine muscle fiber structure^13,18,19^. However, it always requires external labels, which may be harmful and prone to bleaching. More importantly, single muscle fibers with diameters exceeding 50 µm must already be considered thick samples where photon scattering and absorption can become substantial within the illuminated z-cone. An elegant alternative is multiphoton microscopy where infrared light ensures deeper penetration depths and less scattering while laser pulse excitation ensures excitation only within a focal volume of ∼ 1 µm^3^. Using a special nonlinear mode of multiphoton excitation through *second harmonic generation* (SHG), intrinsic signals can be visualized label-free. Biomolecules susceptible for SHG comprise collagen-I and myosin-II, enabling label-free, detailed structural analysis of subcellular cytoarchitecture and myofibrillar geometry in 3D^3,20,21^. Furthermore, several mathematical analysis strategies have been developed to describe the degree of myofibrillar disarray using quantitative morphometry in 2D images^14,20,22^ and in 3D volumes of muscle or single fiber SHG stacks^23–25^. Our group introduced boundary tensor orientation analysis of myofibrillar striation patterns to extract two very sensitive parameters of ‘myofibrillar disorder,’ namely, cosine angle sums (CAS) and vernier densities (VDs). This approach has successfully been employed to describe alterations in 3D cytoarchitecture associated with various disease models^14,24,26,27^. In particular, such studies enabled the identification of the correlation between structure and morphology for the chronic progression of muscle weakness due to age or inherited myopathies.

However, despite all the progress made in SHG imaging and pattern recognition analysis defining ultrastructural alterations, to date there is still no direct proof of how an altered sterical myofibrillar cytoarchitecture is correlated to an impaired muscle force. To obtain such a calibration, simultaneous measurements of SHG signals and isometric force are required in the same single muscle fiber. Therefore, we combined biomechatronics and optical engineering to engineer a miniaturized biomechatronics system, *MechaMorph*, which allocates an optically based force transducer sensor and length/force-feedback controlled voice coil actuator technology in a multiphoton microscopy-adapted stage. To employ a large range of morphological parameters of myofibrillar alignment and isometric force amplitudes, we used single EDL muscle fibers from wild-type and *mdx* mice. We present, for the first time, detailed correlations of the biomechanical parameters of (i) active isometric force and myofibrillar Ca^2+^ sensitivity, (ii) passive viscoelastic parameters, and (iii) optically SHG-derived morphometry (SHG, VD) in a calibration of structure to force. Using this approach, we propose a quantitative prediction of muscle function exclusively from an optical assessment of the structure, without the need for sophisticated biomechanical recordings. This may be very valuable for diagnostics in the scenario of myopathies in future clinical settings involving volumetric SHG imaging of muscle samples/biopsies.

## Results

### Ultrastructural myofibrillar architecture assessed using *second harmonic generation* (SHG) is a predictor of Ca^2+^- activated specific force and myofibrillar Ca^2+^ sensitivity

**Fig. 1:**
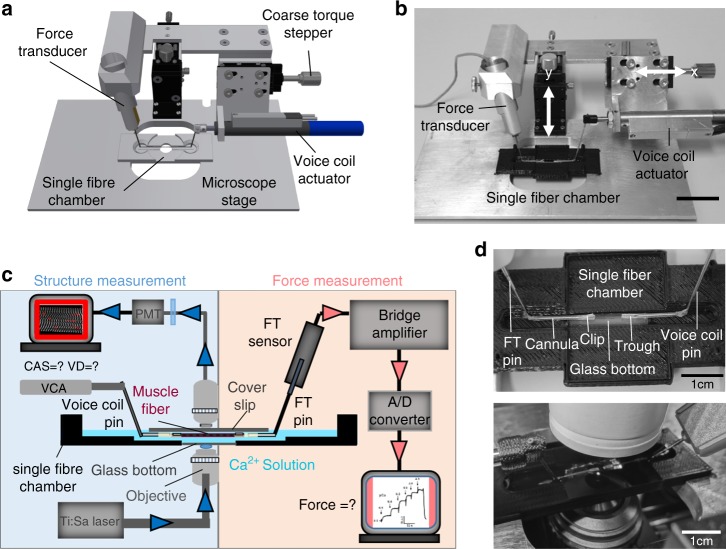
A novel biomechatronics system (MechaMorph) for the simultaneous assessment of isometric, Ca^2+^-activated force and SHG multiphoton imaging in single muscle fibers. **a** 3D CAD sketch of the miniaturized biomechatronics device containing an interchangeable muscle fiber chamber to fit onto a stage of a multiphoton microscope. A force transducer and voice coil actuator pin are connected to a horizontal trough, each fabricated from cannula needles to take up a single muscle fiber (**d**). **b** photograph of the engineered device which is inserted in between two objective lenses to record forward scattered SHG, while the custom-made biomechatronics software runs on a parallel computer (**c**, **d**)

**Fig. 2:**
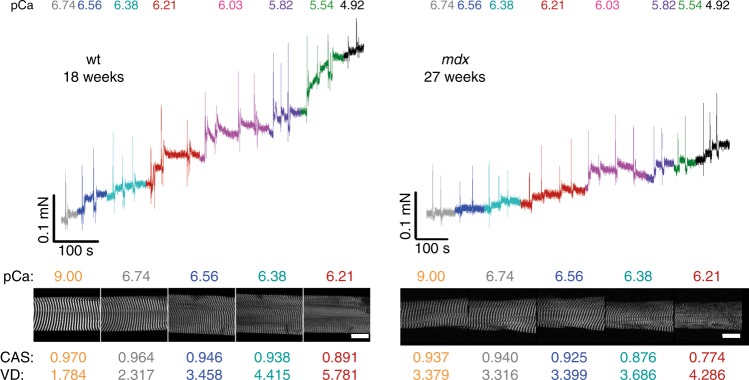
Ca^2+^-activated force and SHG multiphoton imaging simultaneously performed in single wt and mdx EDL muscle fibers. Representative example recordings of force (top) and myosin SHG signals (bottom) from a single EDL fiber from a wt mouse (left) and an *mdx* mouse (right) during successive solution exchange for increasing Ca^2+^ concentrations (decreasing pCa). Brief positive force spikes represent the time points of manual solution exchange performed several times per pCa step. Scale bar: 20 µm. CAS: cosine angle sum (a.u.). VD: vernier density (#/100 µm^2^)

**Fig. 3:**
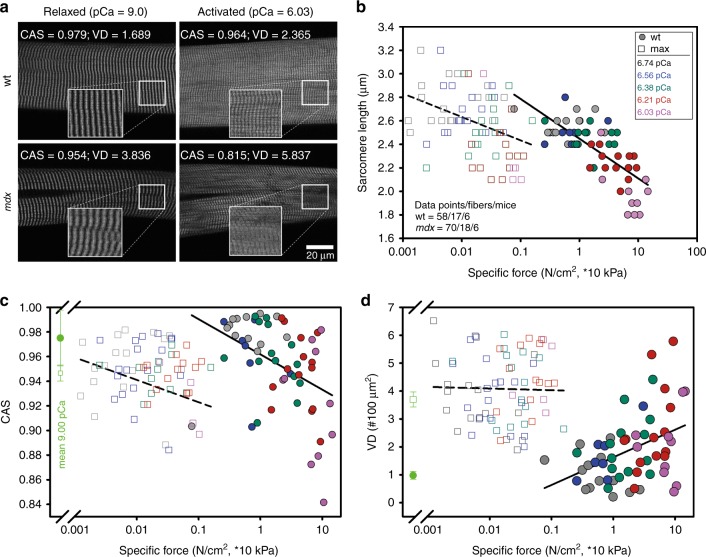
Distortion of sarcomeric and myofibrillar structures in single EDL muscle fibers from both wt and *mdx* mice during active Ca^2+^-induced isometric force generation. **a** representative example SHG images of a single EDL fiber from a wt mouse (17 weeks) and an *mdx* mouse (27 weeks) in the relaxed pCa 9 and pCa 6.03 activated states. During mechanical activation, although isometric, the sarcomere lengths visibly shorten and fiber diameters increase. **b** analysis of the reduction in single fiber sarcomere length during Ca^2+^-dependent force generation (logarithmic plot of specific force) in wt mice (filled circles) and *mdx* mice (open squares); pCa values color-coded. **c**, **d**, changes in myofibrillar/sarcomeric ultrastructural parameters of the cosine angle sum, CAS (**c**), and vernier density, VD (**d**), indicate a Ca^2+^-graded increase in the myofibrillar disorder (CAS↓, VD↑) that is more pronounced in the ordered wt EDL fibers, while single fibers from *mdx* mice are already highly disordered under relaxed conditions

**Fig. 4:**
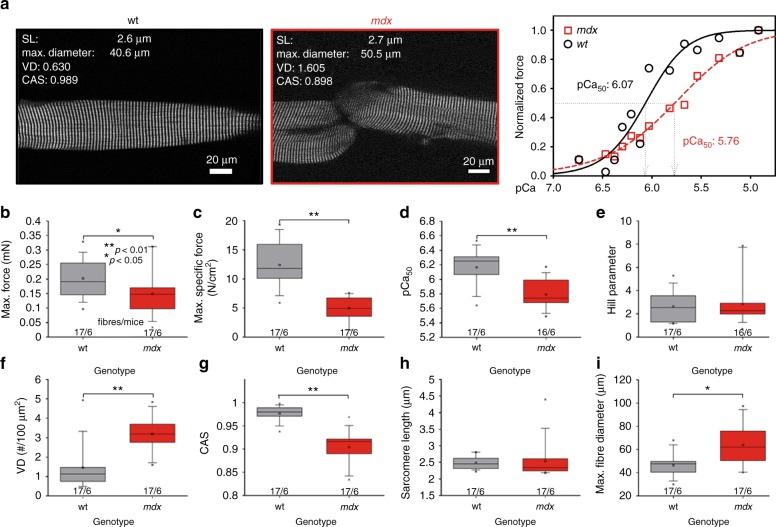
Myofibrillar ultrastructural disorganization in relaxed *mdx* EDL fibers is a predictor of reduced contractile performance and reduced Ca^2+^ sensitivity of the contractile apparatus. **a** SHG images of single EDL fibers from a wt mouse (14 weeks) and an *mdx* mouse (79 weeks) in the relaxed state (pCa 9) with indicated sarcomere lengths (SL), maximum diameters, VD and CAS. Steady-state force at a given pCa normalized to the maximum force with indicated pCa_50_ and Hill parameters for the shown fibers. Single *mdx* EDL fibers showed vast biomechanical deficits in active force production: significantly reduced max. absolute (**b**) and specific force (**c**) at a pCa of 4.92–5.67 and significantly reduced pCa_50_ values indicative of myofibrillar Ca^2+^ desensitization (**d**) with otherwise similar Hill parameters (**e**). This correlates well with myofibrillar structural deficits, as shown by the markedly increased VD (**f**) and decreased CAS (**g**) in *mdx* mice over wt mice. At similar sarcomere lengths (**h**), fiber diameters were larger in *mdx* EDL fibers compared than in wt fibers (**i**). Box plots with box (25 to 75 percentiles), median (line), whiskers (5–95 percentiles), minimum and maximum (x), mean (rectangle) and significances from one-way ANOVA with post hoc Bonferroni test (equal variance) or post hoc Tukey test (no equal variance) indicated as *p* < 0.05 (*) and *p* < 0.01 (**)

**Fig. 5:**
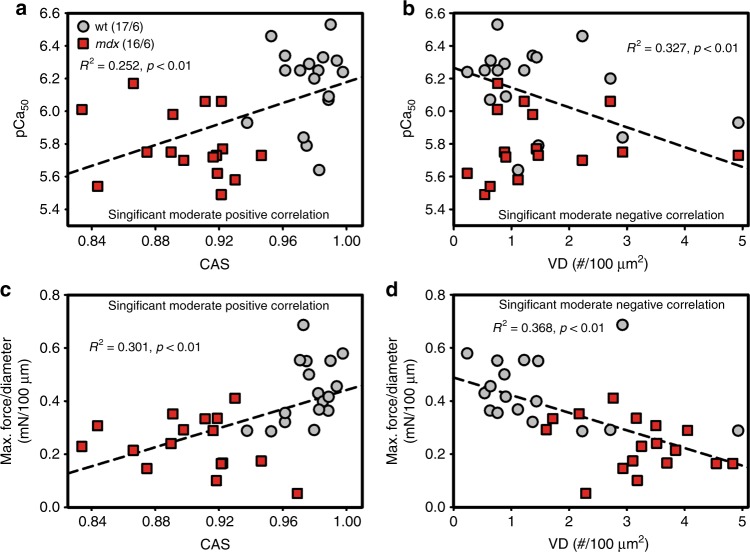
Pearson correlations show significant correlations between SHG-derived structural data and active isometric force parameters in single EDL fibers. Both structural parameters, the CAS and VD, obtained in relaxed (pCa 9) wt (gray) and *mdx* (red) single fibers show significant correlations with pCa_50_ and the maximum force per diameter in the activated state. In particular, the more ordered the myofibrillar arrangement (CAS↑, VD↓), the higher the probability for a higher Ca^2+^ sensitivity (larger pCa_50_) (**a**, **b**), and thus, a higher predicted force production upon Ca^2+^ activation (**c**, **d**). Thus, the CAS positively correlates with both pCa_50_ and the max. force (**a**, **c**), while VD negatively correlates with both (**b**, **d**)

In previous studies, the *mdx* model presented a vastly altered myofibrillar cytoarchitecture, fiber branching and enhanced muscle weakness^12,14,17^. To directly link structure and mechanical function, we sought to calibrate both parameters, SHG morphometry and isometric force amplitudes, within the same single fiber. To exploit a wide range of SHG parameters of CAS, VD, and isometric force, the *mdx* model was chosen to provide values of decreased CAS and increased VD, which are usually not seen in wt single fibers, and to assess the graded isometric force in conjunction with SHG imaging in the same single fiber using our *MechaMorph* system (Fig. 1). Figure 2 shows a direct comparison of isometric force recordings in a single wt and *mdx* EDL muscle fiber that is gradually exposed to solutions with increasing free Ca^2+^ concentrations (decreasing pCa). To complete a pCa step, the solution was exchanged at least three times, as reflected by the force artifacts during the manual exchange. At the end of each pCa step, an SHG stack was recorded, from which the CAS and VD (#/100 µm^2^) were calculated. Figure 2 demonstrates lower isometric force amplitudes in *mdx* mouse samples versus the wt mouse samples and lower CAS and higher VD values throughout the pCa steps. An interesting and somewhat unexpected finding was the increase in VD and decrease in CAS at a higher Ca^2+^ activation  indicative of less ordered myofibrillar alignment, regardless of the underlying genotype. Figure 3 shows analyses of those forces and SHG-derived structural parameters in more detail. First, Fig. 3a shows the SHG images of a wt single fiber and an *mdx* single fiber under the relaxed and pCa 6.03-activated conditions, revealing that the contraction is not purely isometric and that the sarcomere lengths decrease with Ca^2+^ activation due to the finite stiffness of the transducer pin needle. This needle allows deflections at given forces that are detected using SHG morphometry. As seen in Fig. 3b, those deflections are larger in the wt fibers than in the *mdx* fibers due to the larger force production range in the wt fibers. When correlating specific force values from single wt and *mdx* fibers with the CAS and VD over a large pCa range, the Ca^2+^-graded increase in myofibrillar disorder (CAS↓, VD↑), shown in Fig. 2, was fully confirmed with a more pronounced range in the otherwise highly ordered wt fibers under the resting pCa 9 condition compared to the already disordered *mdx* fibers. The *mdx* fibers in particular, started with very large VD values at pCa 9 that were already approximating maximum values and remained fairly constant during the activation with decreasing pCa **(**Fig. 3d**)**. An advantage of the graded pCa activation was the possibility of extracting Ca^2+^-force biosensor curves alongside the SL, diameter, CAS and VD values from the SHG analysis and the corresponding pCa-force curves, as exemplarily shown for wt and *mdx* fibers in Fig. 4a. The sensor curve was right-shifted for the *mdx* fiber, resulting in a lower half-activation pCa, pCa_50_, of the contractile apparatus, which is indicative of a reduced myofibrillar Ca^2+^ sensitivity. This was also confirmed in the group analysis of several single fibers reaching statistical significance for smaller pCa_50_ (Fig. 4d), alongside with significantly reduced maximum force and specific force (Fig. 4a, b), and aberrant ultrastructure (CAS↓, VD↑) in dystrophic fibers (Fig. 4f, g). When combining the wt and *mdx* data to correlate the normalized forces and contractile Ca^2+^-sensitivity with ultrastructural parameters of myofibrillar order (VD, CAS), significant correlations of structure with biomechanical performance were found (Fig. 5). Thus, based solely on the SHG assessment of the ultrastructure of a single fiber, a prediction of the active force is possible.

### Passive stretch impairs myofibrillar alignment by increased axial stress to similar extents in wt and *mdx* fibers

**Fig. 6:**
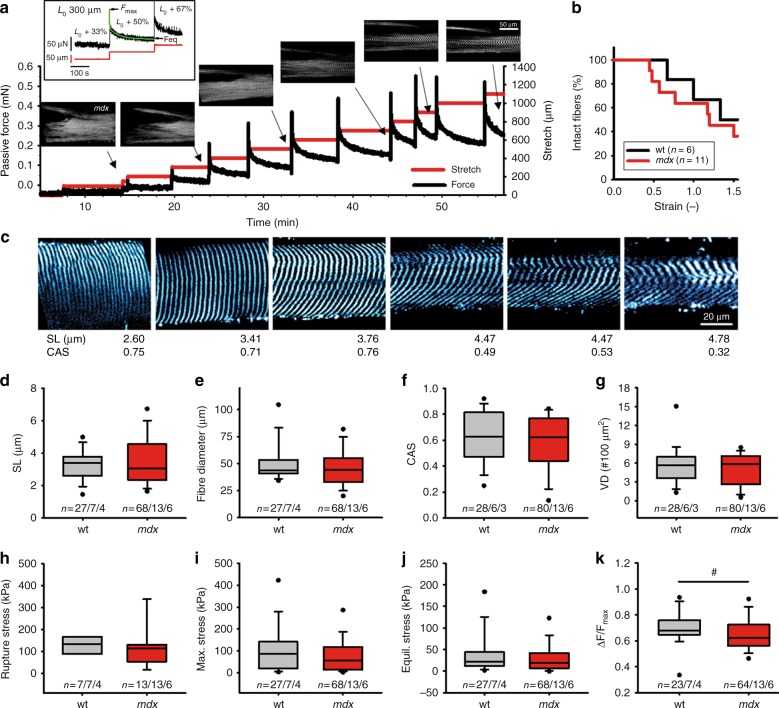
Simultaneous assessment of passive single fiber viscoelasticity biomechanics and SHG myofibrillar ultrastructure in wt and *mdx* EDL muscles. **a** sequence of SHG images (left) taken during a protocol stretching a single EDL fiber from an *mdx* mouse in 50 µm steps and recording restoration force (right). Images were taken at the time points indicated. The inset shows force recording of another *mdx* fiber: instantaneous maximum restoration force *F*_max_ at each stretch was followed by a double-exponential viscous relaxation to a new steady-state elastic force level *F*_eq_. **b** *mdx* single EDL fibers already broke at lower strains compared to wt fibers, compatible with larger stiffness, respective lower viscous relaxation. **c** sequence of SHG images taken from a wt single EDL fiber stretched to the indicated sarcomere lengths (SL) and analyzing CAS values. Note that with the increase in stretch, a marked decline in CAS can be detected as visualized by A-band bending across the fiber cross-section. The SL value ranges were similar in wt and *mdx* fibers (**d**), as were the fiber diameters (**e**), overall CAS values (**f**) and VDs (**g**). Biomechanical passive parameters of the maximum rupture stress (**h**), maximum stress values from all stretches (**i**) and equilibrium strains (**j**) were similar among the wt and *mdx* fibers while the viscous stress relief ΔF/F_max_ values (**k**) were significantly smaller in the *mdx* fibers than the wt fibers, indicating a lower viscosity in the dystrophic genotype. ^#^*P* < 0.025. *n* = (a/b/c) depicts the data from a images on b fibers from c animals

**Fig. 7:**
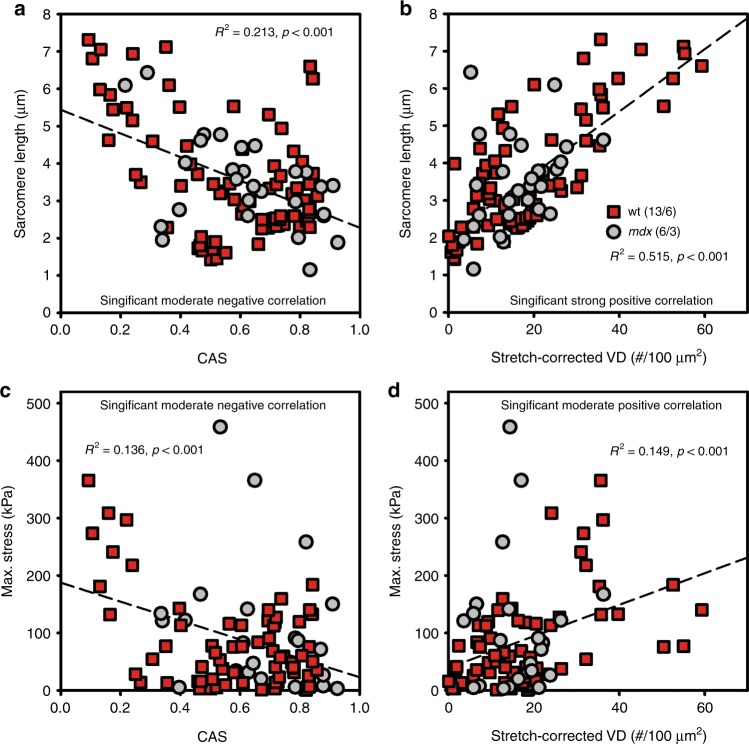
Pearson correlations show significant correlations between the SHG-derived structural data and the passive elastic parameters, as well as a higher degree of myofibrillar disorder with the sarcomere length in stretched single EDL fibers. Both structural parameters, the CAS (**a**) and the stretch-corrected VD (**b**), obtained in relaxed (pCa 9) wt (gray) and *mdx* (red) single fibers show significant correlations with the sarcomere lengths. In particular, the higher the stretch was (SL↑), the less ordered the myofibrillar arrangement became (CAS↓, VD↑). Similarly, the myofibrillar disarray, i.e., the low CAS (**c**) and large VD values (**d**), significantly correlated with increased maximum stress values that occurred during stretching

Apart from assessing active myofibrillar Ca^2+^-activated forces, the design of our *MechaMorph* system also allows assessment of SHG ultrastructural changes with passive strains in single fibers. Figure 6a shows a sequence of recorded passive restoration forces in response to very fast 50 µm step extensions in a single *mdx* EDL fiber and the corresponding SHG image taken at the end of the viscous relaxation phase. That particular *mdx* fiber showed substantial ultrastructural abnormality, e.g., fiber branching. The restoration force pattern always followed an instantaneous elastic restoration force to *F*_max_, followed by exponential viscous relaxation to a steady-state level *F*_eq_, as indicated in the force trace from another *mdx* fiber in the inset of Fig. 6a. The *mdx* fibers were more fragile during step length changes (Fig. 6b), which was probably the result of a significantly reduced viscous relaxation, as indicated by the reduced (*F*_max_−*F*_eq_)/*F*_max_ amplitudes in the dystrophic fibers (Fig. 6k). The sarcomere lengths (Fig. 6d), fiber diameter (Fig. 6e), rupture stress (Fig. 6h), maximum stress (stress corresponding to *F*_max_) (Fig. 6i), and equilibrium stress (stress corresponding to *F*_eq_) (Fig. 6j) were not significantly different between the two genotypes. Intriguingly, when looking at the SHG ultrastructure, ongoing fiber stretch not only resulted in spreading of the sarcomere lengths but, most importantly, in a marked myofibrillar disorder, as reflected by a marked decline in the CAS (Fig. 6c). In particular, there was a prominent bend of the myofibrillar lattice spacing towards the periphery of the fibers (Fig. 6c). Lumping together the CAS and VD values for all stretches did not reveal any difference between the wt and *mdx* fibers (Fig. 6f, g). When stretching fibers, however, one has to keep in mind that with an increasing SL, fewer verniers will remain in the respective field of view. This phenomenon can be compensated for by using a stretch-corrected VD, as described in the Methods. Thus, when correlating CAS and the stretch-corrected VD to the maximum stress values and the SL in all stretched single fibers, Pearson correlations confirmed significant relationships between the SL and the degree of myofibrillar disorder, i.e., a massively declining CAS and an increasing corrected VD (Fig. 7a, b). Since increasing the SL also means increasing restoration forces and thus increasing maximum stress, the resulting correlations for the maximum stress values corresponded to the SL-CAS/-VD_*corr*_ relationships (Fig. 7c, d). Thus, these data confirm a marked myofibrillar disorder with stretch, which was similar in the wt and *mdx* fibers and thus was not related to the presence or absence of dystrophin in single EDL fibers.

## Discussion

### Structure and function studies in skeletal muscle

Apart from organ-tissue-cellular function being regulated on short to intermediate time scales by a plethora of signaling pathways, e.g., involving fast Ca^2+^ signaling or slower G-protein related second messenger cascades, a long-term predictor of cell function is also encoded in the architecture, either within the cells (cytoarchitecture) or affecting the extracellular matrix, ECM (e.g., in fibrosis). In skeletal muscle, genetic, degenerative, or chronic inflammatory diseases have regularly been associated with mechanisms of weakness related to aberrant signaling and structural long-term remodeling, but to date the direct interaction between these two processes has not been determined. This is mostly due to a lack of appropriate technologies that would allow assessment of the structure–function relationship on the single fiber level. While force production in single muscle fibers or even in myofibrils has been studied over the recent decades using various force transducer technologies (e.g., refs ^28–30^), many more studies assessing active forces in muscles are available than those assessing passive viscoelastic behavior. This fact is mostly due to the necessity to manually operate many of the custom-made and commercial systems used to date. The systems usually lack a degree of automation and µm-level precision required for quasistatic steady-state resting-length tension curves or ultrafast and precise stretch experiments to study viscoelastic behavior in single fibers, unlike the more coarse systems used for whole muscle^31^. Therefore, we have very recently introduced a novel automated voice coil actuator-driven system that allows full control of fiber lengths at very slow or fast speeds to record active and passive forces in single muscle fibers^32^. To obtain detailed structural information on the same fiber in situ, we used this concept to integrate our so-called *MechaMorph* into a multiphoton microscopy environment. This unique system allows simultaneous assessment of structure and force, as shown in the present study.

SHG has increasingly been used in recent years to study structural changes in skeletal muscle disease models. In healthy muscle, the regular sarcomere pattern reflected by the aligned myofibrillar architecture can be elegantly exploited by the myosin-origin of skeletal muscle SHG to determine the myofibrillar cytoarchitecture in 3D^3,33^ or even to dissect contractile states of the motor protein interaction^34,35^. In lysosomal storage disease, SHG in conjunction with 2-photon excited fluorescence was used in muscle biopsies from patients with Pompe’s disease or acid α-glucosidase knockout mice to identify intramyoplasmic areas void of SHG signals, representing autophagic debris accumulation^36,37^. In fibers from these models, wavy and pitted myofibrillar patterns were predominant. Since similar patterns, i.e., wavy and disorganized sarcomeres, were also observed in dystrophic muscle, both in sections^20^ and in single fibers^14^, quantitative morphometry approaches were subsequently developed by various groups^20,38^. In addition to Fourier-transform based analyses of sarcomere patterns^38^ or single-frequency wavelet-based Gabor filtering to quantify structural disorder^22^, our group developed pattern recognition algorithms based on boundary tensor analysis into fully automated pattern extraction routines to define two major structural parameters of myofibrillar order, reflected by the CAS (a measure for myofibrillar parallelism) and the density of so-called verniers (a measure for out-of-register appearances of myofibrils)^14,23–25^. In various subsequent age-related studies applying this set of SHG morphometry tools to dystrophic *mdx* or R349P mutant desmin muscle, we established these morphometric parameters for structural diagnosis of ‘myopathy’ and for monitoring disease progression with age^24–27^. Our results using the *MechaMorph* fully confirm the structural CAS and VD parameters in *mdx* single fibers under resting pCa 9 conditions from our previous studies. Moreover, with the advent of combined force-SHG recordings, we can now directly observe the myofibrillar ultrastructure during defined Ca^2+^ activation and during passive stretch. In conjunction with the multiphoton endoscopy system that is currently being developed in our institute, we hope to apply our SHG diagnostics and analysis of muscle tissue in vivo through cannula-based endoscopy in the near future. Even though the backscattered SHG signal will be of lower intensity as compared to transmitted forward-detected signal, we estimate, based on published work, that the signal-to-noise ratio is high enough to conduct morphology analysis^39,40^. Fibrillar molecules such as collagen-I emit coherent SHG in backward direction^41^, as opposed to myofibrillar-based SHG that mainly appears in backward detection due to scattering effects. Polarization-selective analysis of the signal^42,43^ in backward direction might help to further decrease noise in a non-transmission setup.

### Combined SHG morphometry and active biomechanics in single fibers

Although the isometric force levels during maximum Ca^2+^ activation in dystrophic *mdx* fibers in our setting confirmed the findings from the literature of significantly reduced specific forces (Fig. 4)^17,44,45^, a very novel and somewhat unexpected finding was that with increasing myofibrillar tension, myofibrillar disorder increased as indicated by decreasing CAS values and increasing VDs (Fig. 3). This increase in the myofibrillar angular variability was similar to that of *mdx* fibers even in the wt fibers and seems to represent a feature that is unrelated to muscular dystrophy. There is not much information about changes in sarcomere geometries under isometric contractile conditions with graded Ca^2+^ activation. Early snap-freezing and subsequent EM studies in glycerinated fiber bundles from rabbit psoas muscle showed no major changes in the filament lengths of actin or myosin within sarcomeres under rigor, isometric contraction or relaxing conditions^46^. Using an E-shaped tendon force transducer clamped to a *tibialis anterior* muscle of living mice and applying SHG imaging to an electrically stimulated whole muscle in vivo, a vast increase in the sarcomere length variability was seen as a broadened SL distribution, in particular at short muscle lengths (90° ankle) over long muscle lengths (180° ankle)^47^. That very recent study focused on planar images from the top 100 µm and collecting backscattered SHG signals from whole muscle without further details on the 3D aspect of myofibrillar structure under contractile activation; however, it may provide a basis to explain our observed increase in angular disorder of myofibrils if one assumes that lateral forces resulting from different sarcomere lengths of adjacent myofibrils connected by extrasarcomeric proteins, for example desmin, would induce tilts and twists to myofibrils. A study by Nucciotti et al.^35^ also used SHG-imaging of single fibers connected to a force transducer to perform SHG polarization anisotropy (SPA) recordings at different sarcomere lengths under relaxed versus rigor conditions, but not under defined Ca^2+^ activations. Although myosin motor head states could be derived from those SPA analyses, the 3D ultrastructure was not obtained. Thus, our approach represents a more intuitive assessment of myofibrillar alignment, both angular as well as in-register alignment in full 3D with the simultaneous biomechanical assessment of the active and passive force. While VDs increased with the specific force (increasing Ca^2+^) in wt fibers, they remained stationary in *mdx* fibers, probably already reflecting a maxed out upper limit for axial misalignment in this model. The graded Ca^2+^ activation clearly provided a wealth of novel information on not only the correlation between maximum isometric force and corresponding stress to myofibrillar ultrastructure but also on the myofibrillar Ca^2+^ sensitivity which, in our results, was significantly reduced. This may initially appear to conflict with unchanged myofibrillar Ca^2+^ sensitivity in previous *mdx* studies^48–50^. However, in particular in such *mdx* fibers presenting with a high degree of angular variability, the effective contractile Ca^2+^ sensitivity, which is the contractile readout variable in the axial direction, may still be reduced since mechanical fiber activation by Ca^2+^ will inevitably activate force contributions deviating from the main fiber axis^14^, thus requiring higher Ca^2+^ levels to compensate for this angular disorder of the myofibrillar pull. The maximum specific force values assessed in our EDL single fibers of ~10 N/cm^2^ in the wt fibers are well comparable to other studies using single skinned mouse muscle fibers^49^ or fibers from humans^51^. The reduced specific force in dystrophic muscle is, to varying extents, well established in *mdx* mice^17,49^, while in DMD patient *quadriceps* or *biceps* single muscle fibers, the maximum isometric force was not compromised^51^. However, our study was not intended to further elaborate on biomechanics of single *mdx* muscle fibers. Rather, the unique opportunity to assess Ca^2+^-graded force and SHG morphometry in the same fiber motivated us to use the *mdx* model as a tool to obtain SHG-force data sets that we would normally not obtain from healthy muscle fibers, vastly compromised myofibrillar 3D structures and reduced isometric forces. Thus, combining the data sets from single wt and *mdx* EDL fibers enabled us to calibrate the SHG parameters to the isometric force over a large range **(**Fig. 5**)**. These significant correlations now prove what has only been suggested previously: a linear decline in force and Ca^2+^-sensitivity of the contractile apparatus with myofibrillar disorder (indicated by a decreasing CAS and an increasing VD). This is a very new finding, here experimentally verified for the first time. It will open new methods of projecting the force output of muscle fibers, either isolated or within tissue samples, by simply investigating them using SHG imaging.

### Combined SHG morphometry and passive biomechanics in single fibers

Implementing our previous voice coil strategy^32^, also in the *MechaMorph* system, allowed us to perform detailed viscoelasticity assessments on the single fiber level, which is very rare in the literature (for rabbit single *psoas* and *soleus* muscle fibers;^51^ for mouse single *tibialis ant*. muscle fibers^52^). As previously described, sudden stretches are answered by an instantaneous increase in the passive restoration force, followed by a decline in the force due to viscous relaxation of titin filaments^32,51,52^. Our passive stresses are in the range of tens of kPa and increase with stretching, which is in agreement with other studies on single muscle fibers^52^. In our stretch experiments, *mdx* EDL fibers showed similar morphometric parameters to wt fibers, e.g., sarcomere lengths, SHG-derived CAS and vernier densities (corrected for the stretch amount to account for sarcomeres leaving the field of view during stretching), but significantly reduced force relaxation at an unaltered maximum stress, compatible with a preferential impairment of the viscoelasticity over the steady-state stiffness. Additionally, *mdx* fibers broke at lower strains, supporting a compromised passive relaxation. Within the literature, differential results were reported on dystrophic *mdx* EDL muscles either showing no compromise in passive mechanical properties during maturation in mice up to 35 days of age^53^ or in 21 months old *mdx* mice^54^, while another detailed age-related study showed a consistently increased passive stress over a large range of strains in animals aged between 2 and 20 months, compatible with a higher stiffness and compromised viscosity^55^. One reason for the disparate results may be that all those mentioned studies exclusively used whole muscle. Specifically, connective tissue may have a vast impact on passive muscle biomechanics^55^, although one study found that the extracellular collagen content did not alter passive mechanical properties in whole muscles of *mdx* mice^56^. We are not aware of any study on single fiber passive biomechanics in the *mdx* model, and thus our results truly reflect the myofiber viscoelasticity without the influence of ECM components.

Similar to the active force data, the *mdx* model was merely included to extend the biomechanical stress range. However, despite being similar to the wt range, the *mdx* dataset actually expanded the wt dataset toward a higher number of structure-biomechanics pairs for the Pearson correlations, demonstrating, for the first time, that increasing the stretch and thus the passive stress resulted in a vast increase in myofibrillar disorder, as seen by the decrease in CAS and increase in VD to levels beyond those observed in resting fibers, even in strongly abnormal *mdx* fibers^14,24^. The significant linear correlations confirm a direct influence of the mechanical stress on the myofibrillar structure. The prominent drop in the CAS is particularly explained by the observed bending of A-band signals with stretching **(**Fig. 6c**)** that increases the angular distribution of the local force vector orientations and therefore, diminishes the CAS.

In summary, our new combined SHG-biomechatronics approach introduced as the *MechaMorph* system with high precision voice coil technology has enabled us, for the first time, to obtain direct structure–function data pairs in terms of the isometric force, passive viscoelasticity and SHG quantitative morphometry and to establish significant linear correlations between the structure and function at the single fiber level. Thus, we propose that our *MechaMorph* approach adds a further dimension to SHG-derived morphometry resulting in a new noninvasive methodology to predict forces and biomechanical performances in skeletal muscle exclusively using optical assessments, suggesting a translational potential for monitoring disease progression or remission in patients.

## Material and methods

### Animals and single muscle fiber preparation

Adult *mdx* mice carrying a missense mutation in exon 23 of the dystrophin gene (strain: C57BL/10ScSn-*Dmd*^*mdx*^/J) were compared to wild type (wt) mice (C57BL/6N, obtained from Charles River Company). The wt mice were between 13 and 21 weeks of age, and the *mdx* mice were between 27 and 91 weeks of age. We chose that particular age difference because the primary focus was not to compare the differences between the wt and *mdx* phenotypes but to collect a wide range of myofibrillar disorganization and associated impaired biomechanics. In particular, single fibers from older *mdx* mice show reduced CAS levels and increased VD levels unparalleled by adult or aged wt animals. Therefore, the obtained range of optical parameters of the myofibrillar architectures and isometric forces allowed us to correlate both sets of data for a force-SHG calibration. Animal handling was in accordance with the German Animal Welfare Act (Tierschutzgesetz) as well as the German Regulation for the protection of animals used for experimental purposes or other scientific purposes (Tierschutz-Versuchstierverordnung). The investigations were approved by the governmental Office for Animal Care and Use (Regierung von Mittelfranken, Ansbach, Germany; reference number TS-14/2015). All applicable international, national, and institutional guidelines for the care and use of animals were followed. Animals were anesthetized using Isofluorane inhalation. After verifying deep sedation by the absence of a pain reflex when pinching the skin, animals were sacrificed by neck dislocation and both hindlimbs were cut off. The extensor digitorum longus (EDL) muscle was manually dissected in Ringer’s solution, pinned to an elastomer-coated dish and solution exchanged to a ‘high K^+^‘-relaxing solution (HKS, in mM: K-glutamate 140, Hepes 10, glucose 10, MgCl_2_ 10, EGTA 1, pH 7.0) under isometric conditions. Single muscle fiber segments of 2–3 mm length were then dissected in HKS solution through manual tethering of muscle fascicles using fine forceps^26,27^.

### *MechaMorph* biomechatronics system

Our system (Fig. 1) consists of a force transducer element (FT) using in-built optical metrology to measure pin deflection in response to external forces (TR5 S, resonance frequency 550 Hz, range 1.5 µN–0.5 N, compliance 0.7 µm/mN, Scientific Instruments, Heidelberg, Germany), and a software-controlled voice coil actuator, VCA, (SMAC CAL12-010-51BSA, Ispringen, Germany) that allows force- and length-controlled precision feedback positioning^32^. Both the sensor and actuator are aligned in the same plane and are screwed to a steel frame containing one microscrew-driven sledge to lower or lift the apparatus from a single fiber bath chamber clipped into a drilled groove holder on top of the microscope stage plate (Fig. 1a). A coarse micrometer screw allows preadjustment of the x-coordinate along the fiber axis, and the VCA then performs controlled fine-tuned length adjustments in the µm domain (x-position). Fig. 1b shows a photograph of the *MechaMorph* prototype, and Fig. 1c shows a schematic of the system incorporation within the two-objective multiphoton microscope. Both the transducer and voice coil pin were glued to a horizontal trough made from cut-grinding a half-opened polyethylene (PE) tubing (PORTEX SX05, 0.58 × 0.96 mm, A. Hartenstein GmbH, Würzburg, Germany) on each side (Fig. 1d).

### Multiphoton SHG microscopy

Single fibers were imaged using a multiphoton microscope, MPM (TriMScope II, LaVision BioTec, Bielefeld, Germany). A mode-locked ps-pulsed Ti:Sa laser (Chameleon Vision II, Coherent, Santa Clara, CA, USA) was used to excite the SHG signal of myosin-II. The average laser power on the sample was ∼ 16 mW, the pulse duration was ∼ 150 fs and the repetition rate was 80 MHz. A symmetric transmitted light configuration of two water immersion objectives was used for detection. On the excitation side (backscattered, descanned), an LD C-Apochromat lens (40 × /1.1/UV-VIS-IR/WD 0.62, Carl Zeiss, Jena, Germany) was used, and on the transmission side (forward scattered, non-descanned), a W Plan-Apochromat lens (20 × /1.0/(UV)VIS-IR/WD 1.88/DIC M27 75 mm, Carl Zeiss) was used. In the active force experiments, the recorded images had a size of 200 × 200 *µ*m, consisting of 1024 × 1024 pixels, and two to three scans of each pixel were performed at a line frequency of 600 Hz or 1000 Hz and were averaged. The averaging was performed line by line, where the arithmetic mean was taken of the intensity of each pixel. The frame time of a double line averaged image is approximately 4.2 s. With a pixel dwell time of 0.8 µs and a sweep time of approximately 4 µs, the energy that is deposited in each pixel of the sample is 64 nJ, which is approximately three orders of magnitude below the reported damage thresholds^57–59^. SHG signals were excited at 810 nm and detected using a 405/20 nm single bandpass filter (Chroma Technology group, Acal BFi Germany GmbH, Gro¨benzell, Germany) and an ultrasensitive, non-descanned transmission photomultiplier tube (PMT) (H 7422-40 LV 5 M, Hamamatsu Photonics). The linearly polarized excitation light is aligned to 50° or 130° of the fiber orientation^34^ by a rotating half-wave plate, which is located just before the excitation side objective, to maximize the SHG signal intensity. The z-stacks had a step-size of 0.5 *µ*m for recordings in the relaxed state (pCa 9) up to 6.0 µm for recordings at different pCa steps. For the stretch experiments, the z-stack step sizes were 1.0 *µ*m. Image processing and morphometric analysis of the SHG images were conducted as previously described^24,26,27^ to detect *verniers*, i.e., Y-shaped deviations from the sarcomere pattern in a z-stack of the SHG images and to measure the CAS, reflecting the degree of local angular deviation of myofibrillar bundles from the main fiber axis. The normalized VD and the CAS were calculated over fiber z-stacks in the relaxed state before Ca^2 + ^activation (i.e., the relaxed state, pCa 9) and in steady-state of each indicated Ca^2+^ activation (pCa) step. For processing of the SHG image stacks that included vast changes in the SHG intensity with sarcomere lengths^3^ during the stretch experiments (see below), a wavelet-based filter procedure was applied to increase the signal-to-noise ratio^60^. The fiber diameter was obtained by plotting the width of the optical fiber section of each image slice within a z-stack and fitting a Gaussian profile to the data. From the mean fiber diameter, the circular cross-sectional area (CSA) was calculated. The sarcomere lengths (SL) were obtained from the SHG images by thresholding and particle analysis. Specifically, a linear plot in the Fourier domain along the direction of the fiber was created and its peaks were analyzed. The peak with the second highest amplitude was considered to represent the main spatial signal frequency in the main fiber direction, thereby representing the mean SL of the image slice. The mean SL of the entire stack was determined by averaging the SLs over each slice. Slices in which less than 10% of the pixel entries accounted for intensities of at least 10% of the maximum intensity in the stack were not included in the evaluation. To automate the evaluation of the CSA and SL, a macro was written in ImageJ (NIH software, https://imagej.nih.gov/ij/).

### Active, Ca^2+^-activated force recordings

Single EDL fiber segments were mounted between the FT and the VCA pin with the actuator-sensor block lowered into a chamber filled with HKS. The ends of the cut fiber were placed into the troughs and clamp-fixed by clipping in a polyethylene tubing clip. The solution in the chamber was first exchanged for 0.1% (w/v) saponin (Sigma-Aldrich Chemie GmbH, Steinheim, Germany) in a high relaxing solution (HR, mM: Hepes 30, Mg(OH)_2_ 6.25, EGTA 30, Na_2_ATP 8, Na_2_-creatine phosphate 10, pH 7.2) for 20 s to chemically permeabilize the fiber. The fiber was then washed with HR, and the device was mounted onto the adapted MPM stage. By moving the static pin under microscopic control, the sarcomere length (SL) of a fiber was adjusted to 2.2 – 3.1 µm and a z-stack of the fiber was imaged to capture the structure in the relaxed state (pCa 9). The Ca^2+^-activated force was assessed by successively bathing the fiber in solutions with successively decreasing pCa and recording the force until steady-state levels were reached. The solution exchange at each pCa was manually performed using Eppendorf pipettes as that version of the *MechaMorph* system did not yet contain a microfluidics control system (engineering in progress). The maximum activation was measured at a pCa of 4.92 in an undiluted highly activating solution (HA, mM: Hepes 30, Mg(OH)_2_ 6.05, EGTA 30, CaCO_3_ 29, Na_2_ATP 8, Na_2_CP 10, pH 7.2). Specific force was calculated using the fiber diameter in the z-stack taken at the respective pCa. Conducting a sigmoidal Hill fit to the data yielded the Ca^2+^-sensitivity of the contractile apparatus in each single fiber (pCa_50_) and the slope of the sensor curve.

### Passive viscoelasticity recordings during step-stretch experiments

Single EDL fiber segments were clamp-fixed between FT and VCA pins using a PE tubing clip while submerged in an HKS solution. After recording the initial clamping length as a baseline, the fiber was stretched stepwise by moving the VCA using custom-written LabView software. This involved initial stretches of 50–100 µm, eventually up to 400 µm, if the fiber was initially slightly slack and thus, the restoration forces were still low. After each step of length change, the restoration force *F*_R_ was continuously recorded until, after its initial instantaneous increase to a maximum value *F*_max_, the following relaxation phase reached a steady-state of force decline (*F*_eq_, at least 5 min to ensure steady-state). Then, an SHG 3D image stack of the fiber was recorded before proceeding to the next stretch step. Axial fiber strain was obtained from the current VCA position. *F*_max_ was automatically determined from the min–max analysis of the force trace during each step in which *F*_eq_ was extracted from an exponential fit to the force decline during holding of the fiber at the given stretch. The force values were converted to stress using the CSA extracted from the SHG image stacks.

### Statistical analysis

For data comparison, a one-way ANOVA analysis (Sigma Plot, Systat Software) was applied on the two genotypes, wt and *mdx*, with a post hoc Bonferroni test (equal variance) or post hoc Tukey test (no equal variance) where indicated. *p* < 0.05 was considered significant (*), and *p* < 0.01 was considered highly significant (**). The normality of data was tested using the Shapiro–Wilk test. Data are presented as box plots (median value: line, quartiles: whiskers 5–95 percentiles, minimum/maximum values:x, mean: rectangle). Pearson correlations were calculated using the online tool http://www.socscistatistics.com/tests/pearson/Default2.aspx, or SigmaPlot.
